# The Probable Use of *Genus amaranthus* as Feed Material for Monogastric Animals

**DOI:** 10.3390/ani10091504

**Published:** 2020-08-26

**Authors:** Tlou Grace Manyelo, Nthabiseng Amenda Sebola, Elsabe Janse van Rensburg, Monnye Mabelebele

**Affiliations:** 1 Department of Agriculture and Animal Health, College of Agriculture and Environmental Sciences, University of South Africa, Florida 1710, South Africa; manyelo.t.g@gmail.com (T.G.M.); sebolan@unisa.ac.za (N.A.S.); Dutoie@unisa.ac.za (E.J.v.R.); 2 Department of Agricultural Economics and Animal Production, University of Limpopo, Sovenga 0727, South Africa

**Keywords:** amaranth, protein, poultry, amino acids, energy

## Abstract

**Simple Summary:**

In monogastric production, feeds account for about 50–70% of the total costs. Protein ingredients are one of the most expensive inputs even though they are not included in large quantities as compared to cereals. Monogastric animal industries are faced with a major problem of limited protein sources, moreover, the competition for plant materials is expected to further increase feed prices. Therefore, to tackle this problem, interventions are required to find alternative and cost-effective protein sources. One identified crop that meets these requirements is amaranth. Studies have shown the potential and contribution that amaranth has as an alternative ingredient in diets for monogastric animals. Therefore, the main purpose of this review is to provide a detailed understanding of the potential use of amaranth as feed for monogastric animals, and further indicate processing techniques are suitable to improve the utilization of grain amaranth and leaves. Amaranth has shown to improve performance and health status of monogastric animals because of its high nutritional value and availability of phenolic compounds. The conclusion of this review provides evidence from which stakeholders such as feed manufacturers and farmers can use to make more informed decisions in relation to the use of amaranth as an ingredient in monogastric animals.

**Abstract:**

This review presents, discusses, and provides a comprehensive understanding of the potential use of amaranth as feed for monogastric animals. Amaranth is an ancient nutritious crop that has been cultivated for multiple purposes. In America, Asia, and Africa, the leaves of amaranth species are used as vegetables. The change in climatic conditions globally has resulted in shortages of rainfall, unpredictable weather, and lack of inputs such as fertilizer. This has led to scarcity of protein sources in the market and instability in prices which makes it necessary to consider alternative ingredients in poultry, pigs, fish, and rabbits feed formulation. Amaranth is rich in fiber, proteins, vitamins, minerals, and phenolic compounds which have some health benefits in animals and can be used to improve productivity. It also contains anti-nutritional factors which can be reduced by several processing methods. Moreover, its use in monogastric nutrition is useful because amaranth has shown to improve monogastric productivity without having any adverse effect on animals’ productivity. Thus, from this review, it can be concluded that amaranth leaves and grains can be used successfully in monogastric animals though different processing methods which might need to be employed in order to reduce anti-nutritional factors before use in animals.

## 1. Introduction

Amaranth originated from Latin America and is regarded as one of the ancient crops of the world. Its cultivation dates back as far as 6700 BC [1] and there around 60 species of *genus* amaranth of which most are cultivated as grains, leafy vegetables, ornamental plants, and weeds. Amaranth grains have been recorded to be important as feed and food in most countries such as Africa, India, China, Southeast Asia, Mexico, South and North America. Currently, in many tropical and temperate regions, amaranth is extensively grown as a green, leafy vegetable. Several authors have reported that the main species used as grains are: *A. cruentus*, *A. caudatus*, and *A. hypochondriacus* which are also known to be dual purpose varieties [2,3]. However, popular leafy species are *A. hypochondriacus*, *A. tricolor*, *A. hybridus*, and *A. blitum* as described in Table 1 [2,3,4,5,6]. Amaranth belongs to a group of plants in the same genus that are considered to be an annual to short-lived perennial and can be subdivided into grain and vegetables. Compared to soybean, amaranth is a drought resistant crop and has high tolerance to arid environments, although prolonged dry periods induce flowering and decrease leaf yields [7,8]. One of the most important traits that helps this crop to survive in extremely dry conditions is its ability to wilt temporarily and revive immediately after rainfall occurs. It has low capacity for water utilization, resulting in the crop not being able to withstand waterlogging [1]. In places such as Africa, India, and Nepal, amaranth grains and leaves have been established for food use. Recently, amaranth has gained a lot of interest because it is a highly nutritious food crop with excellent nutritional value in both grains and leaves [9]. In addition, it contains secondary metabolites such as phenolic compounds which are well-known as antioxidants found in leaves and other aerial parts. Flavonols, hydroxycinnamic acids, and benzoic acids were identified in amaranth leaves, flowers, and stalks. These compounds play a role in defending against several diseases, such as cardiovascular disease, cancer, cataracts, atherosclerosis, retinopathy, arthritis, emphysema, and neurodegenerative diseases in human beings [10,11,12,13]. Several studies have been conducted in monogastric animals [14,15,16,17,18]. According to Longato et al. [15], 10% of amaranth leaves inclusion level in broiler diets has been found to be more suitable in facilitating better performance characteristics. Whilst [16] reported decreased final body weight when broiler chickens were fed diet with 50 and 100 g/kg of amaranth grain. However, no differences were observed in live weights of broiler chickens fed heat-treated and raw amaranth grains. [17]. Moreover, [18] reported that weight gain or live weight, health status of rabbits were not affected by 160 and 320 g/kg amaranth inclusion in rabbit diets. Whereas, [18] reported an increase in protein and fat contents and a decrease in meat moisture with increasing level (0, 16, and 32%) of amaranth in rabbit diets. In pigs, high in vitro dry matter (DM), crude protein, and energy digestibility were observed when *Amaranthus hybridus* plants and another 19 forage plants were fed to pigs [19]. Therefore, the purpose of this review is to (1) provide a detailed nutritional composition and the benefits to monogastric livestock, (2) summarize the phenolic properties found in amaranth grains and leaves, as well as their contributions to health or as anti-nutritive factors in monogastric animal feeding, and (3) demonstrate the potential use of amaranth grain as source of protein in monogastric diets.

## 2. Methodology

This review mainly focuses on the possible use of amaranth leaves and grains in diets for poultry, fish, pigs, and rabbits. This is because protein ingredients for monogastric animals under intensive systems are one of most costly inputs. Thus, strategies to mitigate these costs associated with feed ingredients have been the core objective of many researchers over the years. Given that this is a review, it was imperative to study most of the available literature in this area of the possible use of amaranth as a protein source. The articles used for the review were attained from peer-reviewed journals, books, and conference proceedings published between 2003 and 2020 in various databases. The length of years was chosen to be able to capture as wide range of articles as possible. The range of keywords and phrases used to search each database included: ‘amaranth feeding in animals’, ‘phenolic compounds in amaranth’, ‘nutritional composition of amaranth’, ‘processing methods of grain amaranth and leaves’. This was done analytically by focusing attention on a mixture of the words and phrases, therefore, authors searched in databases such as Scopus, Web of Science, AGORA, ScienceDirect, and Google Scholar. In the first step of elimination, the search process was conducted using the Google Scholar search engine, where 18,400 studies were listed when amaranth feeding was used as a keyword. The overwhelming majority of these references were evaluated as irrelevant from the point of view of our study. The search was narrowed down by adding in “monogastric”, whereby 675 references were obtained. Furthermore, we narrowed our search with time scale 2003–2020 and we obtained 544 and examined solely on basis of the title and keywords. After the elimination of 483 studies were excluded for differing reasons, 61 full-text remaining studies were assessed for eligibility. The remaining additional literature was included from the examination of the reference lists in the list of the literature extracted, academic resources (Master’s and Ph.D. dissertations), PLoS ONE, and the Directory of Open Access Journals.

## 3. Nutritive Value of Amaranth Leaves and Grains

Amaranth proves to be one of the best-suited crops to address specific health problems globally such as defending against several diseases, like cardiovascular disease, cataracts, atherosclerosis, retinopathy, arthritis, emphysema, and neurodegenerative diseases [14,15,16,17,18]. It contains relatively high nutritional value compared to other exotic vegetables such as asparagus, broccoli, celery, parsley, spinach, etc. Proximate composition of leaves and grains of amaranth vegetables are presented in Table 2. Amaranth grain contains total carbohydrate slightly lower than in common cereals [8,22,23,24,25]. Carbohydrate has the main component known as starch located in perisperm cells and constituting about 48–69% of grain dry matter [25]. Thus, amaranth grains have higher contents of carbohydrates than amaranth leaves. Monogastric carbohydrates serve as an energy provider essential to carrying out normal functions such as growth, movement, and metabolism. Generally, the grains seem to be more superior in nutritive value when compared to the leaves. Several authors reported that amaranth grains have more carbohydrates (51–77%) than amaranth leaves (1.3–10.1%) [26,27,28]. Variation of reported different carbohydrates levels in amaranth grains and leaves might be because reported amaranth was grown at different climatic conditions, whereas amylose content is very low, from 0.1 to 11.1%, in the grain. However, amaranth grains have shown slightly lower dietary fiber than in wheat but have similar dietary fiber like quinoa and common cereals [29,30,31]. According to Arendt and Zannini [23], dietary fiber in amaranth range between 9.8 and 14.5%, depending on the variety. Amaranth is also rich in sugars glucose, galactose, fructose, maltose, raffinose, stachyose, and inositol and is sucrose dominant [22,25].

Several authors reported higher contents of crude fiber in amaranth grains (1.8–6.5%) than in leaves (1.7–3.0) [22,28,32]. Crude fiber in the animal body is responsible for laxation, decrease in blood cholesterol and glucose, as well an increase in digestion [33]. Recently, crude fiber has gained special attention due to its functional value in improving monogastric animal’s gut health [34]. According to [26], improved gut health is important to enhance feed efficiency, promote growth performance, and maintain the overall health of monogastric animals.

Amaranth leaves have a higher moisture content than the grains [22,32,35]. These authors stated that high moisture content in vegetables is an indication of freshness, perishability, and low fats [22,36]. As stated by Venskutonis and Kraujalis [8], Peiretti [37], amaranth grains have shown to have a higher protein content than other cereal grains with more lysine than soybean. Its protein is also relatively abundant in the sulphur-containing amino acids, which are usually limited in the pulse crops [15,16]. In monogastric animals, protein is responsible for growth, reproduction, and repairing of worn out tissues. According to Abolaji et al. [28], Emebu and Anyika [35], vegetables, such as amaranth, have been shown to be rich in protein. Their leaves have lower nutrient composition than grains. Kelly et al. [22], Nicodemas [32] reported that amaranth grains have higher crude protein (13–19.85%) than amaranth leaves (3.37–4.42). This is because seeds play a major role in the growth, reproduction, repairing of plants torn out tissues, and nurturing plant growth, while leaves are responsible for biochemical energy and photosynthesis [26,27]. Unlike with other cereals, amaranth proteins consist of albumins (about 40%), glutenins (25–30%), and globulins (20%) and contain very small amounts of prolamins (2–3%) [23,28,29]. Amaranth prolamins are richer in glutamic acid and essential amino acids than albumins and globulins. This makes amaranth a good protein source closer to the composition of animal proteins [23,29,31].

Crude fats are not really noted to contribute more to the fat supply in animal feeds. However, amaranth grains have a higher fat content (5.7–16.7%) than amaranth leaves (1.79–2.15%). Variation in this variation might be that grains store fats reserve which help in seedling germination. Fats help produce body heat or energy, functioning of the brain, and mobilization and carry fat-soluble vitamins in animal body. Moreover, animal body needs essential fatty acids to perform and enhance immunity [26].

Amaranth grains are richer in ash content than leaves, and according to Abolaji et al. [28], ash content indicates the mineral content present in the feeds, as well high molecular weights. Hence, amaranth grains have a higher ash content than the leaves. However, according to Okpara et al. [38], amaranth leaves contain adequate minerals, such as calcium, phosphorus, and vitamin C in contrast to other vegetable leaves. In addition, Neudeck et al. [39] reported high quantities of both micro and macro nutrients in the seeds and leaves which are associated with preventing health problems such as anemia, lameness, stiff legs, osteoporosis, or rickets, etc. The abundant and availability of nutrients present in both leaves and grains of amaranth as indicated above should put this crop in the front seat of probable alternative sources of protein. Not only is this crop easily accessible but also affordable, which makes it suitable to address high feed costs associated with conventional feed ingredients.

**Table 2:** Comparative proximate analysis (%) of *Amaranthus* leaves and grains.

Nutrient	Leaves	Grains	References
Moisture		6.49	[26]
		6–9	[28]
	72.29		[40]
	79.29		[41]
	86.523	9.643	[27]
Crude protein		16.83	[8]
		19.85	[26]
		13–18	[28]
	4.37		[38]
	4.420	14.753	[29]
	3.7	-	[40]
Crude fat		10.6–16.7	[40]
		5.77	[8]
	1.79		[26]
		6–8	[28]
	2.15		[40]
	1.470	8.473	[27]
Crude fiber		2.5	[8]
		1.81	[26]
		4–14	[28]
	3.01		[38]
	1.757	6.557	[27]
Ash		2.90	[8]
		2.25	[26]
		3–4	[28]
	1.52		[41]
	2.737	2.373	[27]
Carbohydrates	1.337	51.640	[27]
	10.19		[41]
		63	[28]
		77	[26]

## 4. Phenolic Compounds Present in Amaranth Species

Polyphenols are secondary metabolites that occur naturally in cereals, fruits, and vegetables such as amaranth [41]. These compounds vary among *Amaranthus* species and genotypes and they are normally present in various part of plants; seeds, leaves, stalks, and flowers [42,43]. Li et al. [44] found that seeds have higher total phenolic compounds than leaves in different three *Amaranthus* species (*A. hypochondriacus*, *A. caudatus*, and *A. cruentus*). Whereas, Karamać et al. [43] found higher total phenol in the early vegetative stage than in the flower and seeds. Studies have shown that the amaranth crop has natural antioxidant phytochemicals that help in defending against cardiovascular diseases [11,45,46]. Antioxidants found in amaranth have been reported to be more powerful than those found in Vitamin C, E, and beta carotene. According to Jimenez-Aguilar and Grusak [46], antioxidant activity includes suppression of reactive oxygen species (ROS), such as peroxides and superoxide formation, by inhibiting enzymes and destroying free radicals.

Several studies have reported that amaranth contains phenols compounds such as rutin and quercetin [3,42,43,47]. According to Kalinova and Dadakova [48], *A. tricolor* flowers contained lower amounts of rutin than leaves. Rutin and quercetin have shown to be important antioxidants which inhibit the oxidation of high-density lipoprotein cholesterol [48]. High phenolic acids in leaves, flowers, stalks, and seeds of *A. caudatus* contain high phenolic compounds [44,49]. Moreover, stems of *A. spinosus* were reported to contained a lot of Coumaroylquinic acids [50]. Whereas, Stintzing et al. [51] reported caffeic acid and vainillic acid as the principal antioxidant compound found in leaves extract of *A. caudatus*. Moreover, high contents of gallic acids was reported in the seeds and sprouts of *A. cruentus* by [8]. However, free phenolic acids, such as ferulic, *p*-coumaric, *p*-hydroxybenzoic, vanillic, sinapic, gallic, and protocatechuic acids, as well as betacyanins, were not detected by [44] in their study of antioxidant activity and phenolic composition of amaranth (*Amaranthus caudatus*) during plant growth. Thus, amaranth phenolic compounds content might depend on amaranth species and their various plant parts (seeds, leaves, flowers, stalks, stems, etc.). The antioxidant properties of polyphenolic compounds present in amaranth may be directly used in animal nutrition. They can be applied as preservatives in compound feeding stuffs to protect animals against the negative consequences of oxidation of feed components, feeds with a high content of unsaturated fat.

## 5. Anti-Nutritional Factors Present in Amaranth

Ref. [52] defined anti-nutritional factors (ANF) as “those substances generated in natural food stuffs by different mechanisms (e.g., inactivation of some nutrients, diminution of digestive process, or metabolic utilization of feed) which exert effect contrary to optimum nutrition”. Amaranth contains secondary metabolites, such as tannins, oxalates, phytates, trypsin inhibitors, saponins, and nitrates (Table 3). These anti-nutritional factors are biologically active chemical compounds which depress the availability of nutrients to the animal body and interfere with metabolic processes [8,14,52]. Tannins are condensed bitter plant polyphenolic compounds that are present in high amounts in the seed coats of most legumes, and certain fruits and vegetables, including the amaranth [53]. These compounds are distributed throughout the plant kingdom and are common in both gymnosperms and angiosperms. Tannins have traditionally been considered anti-nutritional, but it is now known that their beneficial or anti-nutritional characteristics depend on their chemical structure and dosage. The most abundant tannins are condensed tannins [54] and these can inhibit digestion by binding to plant proteins, making them more challenging to digest [55]. They also interfere with protein absorption and digestive enzymes. Oxalates are known to be anti-nutrients present in high amounts in amaranth vegetables. These secondary metabolites form insoluble calcium oxalate with calcium, and this results in preventing the absorption and utilization of calcium by the body of an animal, hence causing diseases such as rickets and osteomalacia [56]. As stated by Fasuyi et al. [14], phytic acid, also known as phytate when in the salt form in many plant tissues, is the storage form of phosphate which binds iron, zinc, calcium, and magnesium. This phytic acid has been shown to result in insoluble complexes that are normally not easy to be absorbed by a chicken’s gastrointestinal tract. During the germination stage of grains, such as amaranth, the phytate decreases due to enzymatic breakdown of phytate which improves iron availability. Fasuyi et al. [14] reported that phytate content of between 1 and 6% in animal diets decreases the bioavailability of mineral elements, especially in monogastric animals, over a long period. Amaranth is known to be one of the crops that accumulate high number of nitrates. Nitrates in amaranth are a concern most of the time because they may be chemically changed in the digestive tract of the animal and become poisonous. This has been shown to occur mostly when photosynthesis is slowed down by factors, such as herbicides, drought, or frost, and with a high accumulation of nitrogen fertilizer in the soil. This shows that as much as amaranth can contain useful nutrients and phenolic compounds which have been proven to improve performance and health status, it cannot be readily used without undergoing several processing methods to reduce these anti nutrients. Thus, several researchers have tested the different methods suitable for both grains and leaves for use in monogastric diets. What should be key when selecting these methods is their cost-effectiveness, or else it can defeat the purpose of using amaranth as a cheap and readily available source of protein in monogastric feed formulations.

## 6. Processing Methods Used to Reduce Anti-Nutritional Factors (ANF’s)

As already indicated and explained, the presence of the anti-nutritional factors is one of the major problems with alternative feed sources, such as the amaranth crop, which mostly require chemical, biological, or physical treatment before they can be effectively included in monogastric diets. There are several processing methods that are often applied to reduce anti-nutritional factors in vegetables like the amaranth. Some of these processing methods are practised domestically, and others are performed on a larger scale in the industry [61]. To date, many researchers have been working on how anti-nutritional factors can be reduced in animal diets. Cooking, roasting, extrusion, etc., have shown to be one of the mechanical and physical methods that can be used to reduced anti-nutritional factors. The use of alkali treatment, ammonia, urea, polyethylene glycol, and polyvinylpyrrolidone (PVP) have been used as a chemical treatment, and enzyme supplementation has been used as biological methods [62].

### 6.1. Cooking

Cooking is one of the ancient methods that has been used to reduce anti-nutritional factors in animal diets. Crops, such as amaranth, need to be cooked or heat-treated before they can be given to animals because of its high content of anti-nutritional factors. Studies have shown that about 20% or more of raw amaranth cannot be included in broiler diets. However, 40% of amaranth can be included in broiler and layers diets if cooked and will have no adverse effect on production performance [68]. According to Mamiro et al. [69], cooking is beneficial to the nutritional value of feed stuffs because it does not reduce or affect availability of minerals and proteins. Several authors reported that cooking improves nutritional values of various vegetables, and results in a high reduction of phytates and oxalates in green leafy vegetables, such as amaranth [70]. However, other authors reported that phytic acid content is not affected by cooking [71,72]. In studies where anti-nutritional factors were affected by the cooking method, authors stated that the effects might be due to heat degradation, a change in chemical reactivity, and the formation of insoluble complexes [70,72,73].

### 6.2. Roasting

Roasting is one of the heat methods from the past that has been used to reduce anti-nutritional factors. This method is done by bringing feedstuffs such as amaranth in contact with direct flame. The process of roasting has shown good results when done at a temperature of 180 °C. After roasting, mechanical cooling is recommended to bring down the temperature of the feedstuff. According to Chemeda and Bussa [74], the roasting method has proven to decrease the trypsin inhibitor activity, tannins, and phytates in amaranth grains. Moreover, roasting has shown to decrease viscosity, the ability to bind and retain water, and the gelling ability of grains. Agume et al. [75] reported that the decrease in all these properties is significantly important when amaranth grains are roasted [75]. The good thing about this method is that it causes little decrease in protein and carbohydrate content of feedstuffs and provides a good number of vitamins and minerals, while reducing the largest part of tannin, phytate, and trypsin inhibitor activity [76].

### 6.3. Popping

Popping is one of easiest and cheapest methods to reduce anti-nutritional factors, and is normally used for household food. This method is like roasting except that feedstuffs do not come into direct contact with flame but are heated only with hot air. For a successful popping of feedstuff to happen, a temperature between 160–193 °C is recommended, thereafter it is then cooled down gradually using mechanical cooling, as done in roasting. Several authors reported that popping reduces anti-nutritional factors, such as phytic acids in amaranth grains but results in a decrease in protein content which might be because some amino acids are very sensitive to heat [76,77,78]. However, the decrease in phytic acid content during the popping method results in more bioavailability of protein and minerals, and an increased level of carbohydrates [78,79]. According to Muyonga et al. [80], higher coefficients of nutrient digestibility (crude protein, ether extract, Neutral Detergent Fibre (NDF), Acid Detergent Fibre (ADF), and gross energy) were observed when amaranth grains were heat treated using popping method, whereas [81] reported a decrease protein digestibility in broiler chickens fed popped amaranth grains. Juzl et al. [82] reported that better meat sensory indicators (taste, tenderness, texture, and color) in cockrels and pullets fed diets supplemented with 10% of popped amaranth grains.

### 6.4. Extrusion

Extrusion is a heat processing method that requires a high temperature for a short period of time. Furthermore, it is done by a combination of temperature, moisture, pressure, and mechanical shear. According to Nooshin et al. [83], this method involves putting feedstuffs in plastic with water to cook. Authors reported that extrusion has resulted in a decrease in tannins and polyphenols while improving in vitro protein digestibility [84]. According to Kumar et al. [85], an improvement in in vitro protein digestibility is a result of the degradation in protein complexes within the extruded samples and the denaturation of protein due to heat and shearing. Popiela et al. [86] reported that fatty acids in the egg yolk of layers was not affected by supplementation of extruded amaranth grains. Furthermore, authors reported an increase in final body weight in layers fed extruded amaranth grains [86]. Jakubowska et al. [87] found no effects in breast meat of quail supplemented with extruded grains for 14 weeks. In pigs, Sokňl et al. [88] reported that extruded amaranth grains were found to have no effect on meat quality.

### 6.5. Alkali Treatment

Alkaline treatment is an ancient chemical treatment of anti-nutritional factors in animal diets. It can be done by simply adding urea, NaOH, polyvinylpyrrolidone (PVP), and polyethylene glycol (PEG) to reduce anti-nuttiness in animal feeds [89]. Currently, PVP and PEG are more promising chemical strategies in reducing anti-nutrients in animal feeds [90]. PEG is an inactive and unabsorbed chemical that contains oxygen atoms which form hydrogen bonds with phenolic groups present in tannins and destroy them and for stable complex which prevents tannins to bind proteins [60]. Several studies reported that polyethylene glycol can prevent the formation of tannin–protein complexes and transfer pre-formed tannin–protein complexes [60,91]. This has shown to reduce tannins activity and to increase the availability of nutrients, such as proteins, and to reduce microbial inhibition. [90,92] stated that increased availability of nutrients resulted in improved animal performance and neutralized the negative effects of tannins in crops. Alkali treatment has been shown to have beneficial effects in animals depending on the amount of anti-nutrients present in the diets [93,94]. Thus, no studies have been yet reported on the effects on monogastric animal performance fed chemically treated amaranth.

### 6.6. Enzyme Supplementation

Enzyme supplementation is an expensive biological treatment and has shown to outshine processing methods, such as cooking or heating [25,94,95], although it is expensive. Several studies have shown that enzyme supplementation has been effective in vegetable-based poultry diets to reduce tannins [14,96,97]. Enzymes, such as cellulose, glucanase, and xylanase, have been used to reduce anti-nutrients in animal diets because most of alternative feed sources are rich in fiber, protein, vitamins, and minerals. Choct [96] reported that amaranth leaf included in broiler diets has no deleterious effect when supplemented with Roxazyme G2 enzyme containing cellulase, glucanase, and xylanase. Furthermore, these authors reported that up to 25% of sun-dried *A. cruentus* leaf meal supplemented with an enzyme (cellulase, glucanase, and xylanase) can result in better performance of broiler chickens. According to Fasuyi and Akindahunsi [97], Bhardwaj et al. [98], cellulase enzyme is responsible for breaking down β-1,4-glucoside linkages of cellulose chain and converting them into beta-glucose, whereas xylanase enzyme breakdown β-1,4-glycoside linkage of xylan into xylose and glucanase enzyme breakdown a glucan glucose sub-units. Increased nutrient digestibility, a decrease in the incidence of wet litter, and the excretion of nitrogen and phosphorus in broiler chickens have been reported as important benefits of exogenous enzyme supplementation [99,100,101]. Thus, enzyme supplementation can be used in animal diets to improve utilization of available and alternative feed sources with anti-nutritional factors.

Usually several factors are taken into consideration when selecting an appropriate processing method for grain crops. A challenge with most of the non-conventional feed ingredients is the presence of heat labile factors which has led to being classified as growth inhibitory anti-nutritional factors that limit their acceptability and utilization by monogastric animals [86,87,88]. Therefore, since crops like amaranth are termed “cheap” in the quest of reducing feed costs, perhaps more research should be invested in processing techniques which are cost-effective and easy to use.

## 7. The Use of Amaranth Leaves and Grains in Monogastric Nutrition

Amaranth crops are normally grown for leaf and grain production in most parts of the world [68]. Amaranth leaves and grains are a potential source of protein and methionine for monogastric animals. Monogastric nutrition involves providing balance of nutrients that best meets the animals’ needs for growth, maintenance, and egg production. This crop gained global attention due to its availability, economic feasibility, traits of being hardy in harsh environments, and its excellent nutrient composition [17,102]. The inclusion of amaranth, both leaves and grains, in animal feed has shown to be a promising alternative source.

### 7.1. Amaranth Leaves

The use of amaranth leaves has shown improvement in monogastric feed formulations as unconventional sources of energy and protein (Table 4). According to Fasuyi et al. [14], *Amaranthus cruentus* leaf meal could be added to broiler finisher diets as a potential protein source. Authors reported that at least 10% of *A. cruentus* leaf meal can be included in broiler finisher diets and results in better performance. Moreover, 10% of amaranth in layers has shown to reduce the cholesterol content of eggs and improve egg weight [15], while Fasuyi and Akindahunsi [97] reported that up to 25% of sun-dried *A. cruentus* leaf meal supplemented with an enzyme (cellulase, glucanase, and xylanase) can result in better performance of broiler chickens. In fish nutrition, Ngugi [103], in their study of characterization of the nutritional quality of amaranth leaf as a protein source to replace fish meal in Nile tilapia, reported a possible replacement of fishmeal with up to 80% amaranth leaves. Authors reported that an 80% replacement of fish meal with amaranth meal had no effect on the performance of tilapia fish. However, Adewolu and Adamson [12], Punita and Chaturvedi [104], Phonekhampheng [105], observed an improved growth rate of fish fed up to 5% amaranth leaves. Adeniji et al. [106] reported a reduced growth rate of fish at all inclusion levels of up to 75%. Authors stated that the reduced growth rate might be due to the highest level of amaranth with high content of anti-nutritional factors and imbalanced amino acids (Adeniji et al. [106]).

### 7.2. Amaranth Grains

Amaranth grain has the potential to partially replace maize and soybean meal in poultry diets (Table 5) [68]. The grain has an adequate amino acids content which resulted in the grains having about twice the amount of protein as cereal grains. They also have a similar energy content as other cereals, thus, they can be used as a feed ingredient for broilers. Authors recommended that amaranth grains can only be included as a raw material in chicken finisher diets [9]. However, other authors have normalized treating grains before consumption by chickens. In fact, heat treatment, such as autoclaving, extrusion, cooking, or popping of amaranth grains, gave positive results compared to results from feeding maize/soybean rations [110]. Písaříková et al. [81] used popped amaranth grains which resulted in lower in vitro digestibility of protein than with raw amaranth. This was due to the high temperatures that resulted in a decreased biological value of protein. However, Fasuyi and Akindahunsi [97] stated that heat treatment of amaranth grains resulted in lower digestibility than raw amaranth in broilers. According to Ravindran et al. [111], amaranth grains can be incorporated at levels of up to 400 g per kg without adverse effects as a potential useful energy supplement for broiler diets. Popiela et al. [86] indicated that a greater egg production was observed when layers were fed with amaranth grains. Currently, in pig nutrition, there is limited literature on the use of amaranth grains. The use of amaranth in pig nutrition has shown positive results in growth performance. Several authors reported an increase in digestibility when amaranth is added to pig diets [18,108]. However, 2.5 and 10% of *Amaranthus cruentus* and grass meal can be used to increase the production and survival of weaner pigs [108]. Niewiadomski et al. [112], in their study to demonstrate the usefulness of amaranth as a component of commercial feeds for rainbow trout, observed an improved growth rate of trout fish when 5% of soybean meal was replaced with amaranth meal. *Amaranthus dubius* is proposed as a possible alternative source of fiber and protein in rabbit feeding for subtropical and tropical regions [17]. Authors reported that there was no adverse effect observed in weight gain and health status of rabbits fed a diet with *A. dubius.* However, feed intake, feed conversion, and digestibility were improved in diets containing or supplemented with *A. dubius* [17]. Molina et al. [17] reported an increase in protein and fat contents, with a decrease in moisture content of rabbit meat when *A. dubius* was included in rabbit diets. Nicodemas [32] reported that treated amaranth grain inclusion in rabbit diets can revert an associated endothelial dysfunction, lower tissue and blood cholesterol oxidation in rabbits.

### 7.3. Effect of Amaranth Grain and Leaves on Health Parameters of Monogastric Animals

Amaranth has antioxidants which makes a candidate of potentially improving health status of monogastric animal. The health properties of diet supplements are proved by, among other things, the values of hematological and biochemical blood indices. Several authors reported the effect of amaranth inclusion on health parameters of monogastric animals. Sokňl et al. [88] reported that amaranth grains supplemented in pig diets resulted in blood glucose reduction. According to Fasuyi [117], broiler chickens fed finisher diet supplemented with amaranth leaves showed no predisposition to acute general infection, malignant tumor, malformation, or diseased condition even at the highest amaranth inclusion level of 20%. Moreover, Rouckova et al. [16], Orczewska-Dudek et al. [113], reported that blood plasma glucose level of broiler chickens fed amaranth grains up to 8% were not affected. However, Czerwińska et al. [118] observed an increase in plasma lipid profile of rats fed amaranth grains. Blood plasma triglyceride level was shown to decrease by inclusion of amaranth in Japanese quails [119]. The reduction of blood plasma triglyceride was closely dependent on the content of bioactive substances, including antioxidants in amaranth seeds [119]. It can be assumed that the addition of amaranth seeds can have health benefits in monogastric animals, although more research is recommended in this area.

## 8. Conclusions

This review describes the use of amaranth grain and leaves as an alternative feed source in monogastric animals. Various researchers found that amaranth can partially or completely replace soybean or fishmeal in the formulation of feed for poultry, fish, pigs, and rabbits. Reasons highlighted by most researchers were that amaranth has less competition with soybean for human consumption and can grow in harsh weather conditions where soybean does not do well. Moreover, amaranth contains phenolic compounds which depend on species and various plant parts (seeds, leaves, flowers, stalks, stems, etc.) which are beneficial to human and animal health alike. It is recommended that grains be subjected to different processing methods to reduce anti-nutritional factors before use in monogastric animals.

## Figures and Tables

**Table 1:** The origin, type, and uses of amaranth species worldwide.

Amaranth Species	Origin	Leafy or Grain	Uses	References
*A. cruentus*	America	Grain and leafy vegetable	Food, medicine, ornamental	[19]
*A. thunbergh*	Africa	Leafy vegetable	Medicine, fodder for livestock	[2,20]
*A. spinosus*	America	Leafy	Medicine, food	[20]
*A*. *graecizans*	Africa	Leafy	Food	[20]
*A. hypochondriacus*	America	Grain and leafy vegetable	Ornamental, medicine	[20,21]
*A. dubius*	America	Leafy vegetable	Medicine	[2,20]
*A. tricolor*	America	Grain and leafy vegetable	Medicine, ornamental	[2,20]
*A. blitum*	Africa	Leafy vegetable	Medicine	[2,20]
*A. hybridus*	America	Leafy vegetable	Food, medicine	[6]
*A. lividus*	America	Leafy vegetable	Food	[5]
*A. caudatus*	America	Grain and leafy vegetable	Medicine, ornamental	[3,19]

**Table 3:** Key anti-nutritional factors found in amaranth.

ANFs	Adverse Effect	References
Phytic acids	Affects bioavailability of calcium and other micronutrients, such as iron, copper, and zinc	[57,58,59]
Tannins	Form complexes with proteins that in turn cause inactivation of many digestive enzymes and decrease protein digestibility	[59,60]
Oxalates	Limit food mineral bioavailability	[58,59]
Enzyme inhibitors	Reduce protein digestibility and retard growth	[59,61,62,63]
Saponins	Inhibitory activities of digestive enzymes, such as amylase, glucosidase, trypsin, chymotrypsin, and lipase, which can cause indigestion-related health disorders, result in reduced intestinal absorption of many nutrients	[63,64,65]
Nitrates	Weaknesses and rapid pulse	[66,67]

**Table 4:** Effect of amaranth leaf meal in diets of monogastric animals.

Monogastric Animal	Inclusion Level	Findings	Authors
Poultry	5–10%	Reduces the cholesterol content of the eggs, lowers growth performance, cholesterol, triglyceride, and serum lipid peroxidation levels	[15]
	10–20%	Improved performance characteristics and nitrogen utilization	[14]
		Improved egg weights	
	8%	No adverse effect on live weight, feed conversion, carcass characteristics, and meat quality	[15]
Fish	80%	No adverse effect on performance	[103]
	75%	Reduced growth rate	[106]
	5%	Improved growth rate	[12,105]
Rabbits	20–30%	Reduced feed intake	[107]
	32%	Low growth rate	[15]
Pigs	10%	Increased digestibility, the degree of assimilation of nitrogen, and the productivity of weaners	[108,109]

**Table 5:** Effect of amaranth grain in diets of monogastric animals.

Monogastric Animal	Inclusion Level	Findings	Authors
Poultry	10%	Reduced the cholesterol content of the eggs, lower growth performance, cholesterol, triglyceride, and serum lipid peroxidation levels	[15,104]
	8%	Reduced body weight, increased FCR	[9,113]
	8%	No adverse effect observed on body weight and growth performance	[16]
	5%	Greater egg production and lower feed conversion ratio	[86]
Fish	5%	Improved growth rate	[112]
Rabbits	20%	Lower tissue and blood cholesterol oxidation	[114,115]
	32%	Increased protein and fat contents	[17]
	25%	Negative effects on the growth rate and DM feed conversion ratio	[116]
Pigs	25%	No adverse effect on the chemical composition, physical-chemical, or sensory properties of the meat	[88]
	10%	No adverse effect on health or metabolism	[109]
	2.5–10%	Increased digestibility	[18,108]
